# Negation and social avoidance in language recruits the right inferior frontal gyrus: a tDCS study

**DOI:** 10.3389/fpsyg.2024.1356030

**Published:** 2024-05-03

**Authors:** Enrique García-Marco, Aarón Nuez Trujillo, Iván Padrón, Yennifer Ravelo, Yang Fu, Hipólito Marrero

**Affiliations:** ^1^Department of Clinical and Experimental Psychology, University of Huelva, Huelva, Spain; ^2^Institute of Neurosciences, University of La Laguna, San Cristobal de La Laguna, Spain; ^3^Faculty of Humanities and Social Sciences, Psychology, Technological University of Peru, Lima, Peru; ^4^Department of Evolutionary and Educational Psychology, Faculty of Psychology and Speech Therapy, University of La Laguna, San Cristóbal de La Laguna, Spain; ^5^Departamento de Psicología Cognitiva, Social y Organizacional, Universidad de La Laguna, San Cristóbal de La Laguna, Spain; ^6^School of International Studies, Zhejiang University, Hangzhou, China

**Keywords:** approach/avoidance attitudes, tDCS, verbal understanding, interpersonal cognition, negation, right inferior frontal gyrus

## Abstract

**Introduction:**

In the process of comprehension, linguistic negation induces inhibition of negated scenarios. Numerous studies have highlighted the role of the right Inferior Frontal Gyrus (rIFG) - a key component of the inhibitory network - in negation processing. Social avoidance can be linguistically portrayed using attitudinal verbs such as “exclude” vs. “include”, which inherently carry negative connotations. Consequently, we hypothesize that the interplay between explicit negation and the implicit negativity of avoidance verbs can be modulated via transcranial direct current stimulation (tDCS) targeting the rIFG.

**Methods:**

In our study, sixty-four participants read approach/avoidance sentences, which were either affirmative or negative, such as “Anne included (did not include) meat in her diet” vs. “Anne excluded (did not exclude) meat in her diet”. This reading task followed a 20-minute tDCS session. The sentences were sequentially displayed, and at 1500 ms post-sentence, a verb was shown – either the one previously mentioned or its semantic alternative counterpart (e.g., included vs. excluded).

**Results:**

Findings revealed that anodal stimulation intensifies the inhibitory impact of negation during sentence comprehension. Under anodal conditions, negative sentences led to extended reading times for the mentioned verbs compared to their affirmative counterparts, suggesting an increased inhibitory effect on the verb. Furthermore, in avoidance sentences, anodal stimulation resulted in reduced reading times for alternative verbs (e.g. “included”) in negative sentences compared to alternative verbs (e.g. “excluded”) in negated approach sentences.

**Discussion:**

As “avoidance” is semantically equivalent to “non-approach”, the inhibitory effect of negation is primarily applied to the implicit negation: NOT EXCLUDED = NOT→NOT (INCLUDED), which consequently activates the representation of the alternative verb making it more available. We further discuss these findings in light of the rIFG’s pivotal role in processing attitudinal verbs and linguistic negation. This discussion is framed within the overarching context of the two-step model of negation processing, highlighting its significance in the realm of social communication.

## 1 Introduction

Language is tightly interwoven with interpersonal cognition (Tylén et al., 2010; Seyfarth and Cheney, 2014; Corballis, 2017). Using approach and avoidance verbs, language enables us to represent and communicate our own attitudes, as well as those of others, in social contexts. This helps regulate our social interactions by expressing preferences or aversions (Marrero et al., 2023a,b). In social settings, we express our preferences using attitudinal expressions, often these preferences are indexed by verbs that denote approach or avoidance (Marrero et al., 2020, 2023a,b). Avoidance verbs suggest a negative stance toward stimuli. Early in infancy, we use both bodily and linguistic signs, like head shaking or verb denial, to show our dislike for certain stimuli (Clark, 1978; Austin et al., 2014; Beaupoil-Hourdel et al., 2016). Over time, verbal avoidance action may become represented as “against a stimulus approach”; given that “against” is perceived as negative (Clark, 1978; Xiang et al., 2016), avoidance verbs inherently would be represented as implicitly negative, like “exclude” being understood “not include” (Marrero et al., 2020; see also Dudschig et al., 2023 and Montalti et al., 2024).

Negation is an important linguistic operator (Horn, 1989), which can also serve social communication. Using questionnaires, the role of negation in reversing the meaning of attitudinal sentences from approach to avoidance, and vice versa, has been demonstrated off-line (Marrero et al., 2020). Given its cognitive and social relevance, it would be of interest to examine the brain’s basis for approach and avoidance and the interplay with negation in verbal understanding for interpersonal cognition.

The right Inferior Frontal Cortex (rIFG) has been shown to be involved in inhibitory processes, both motor and cognitive (Aron et al., 2014). Since linguistic negation induces inhibition of the negated state of affairs, such as “an open door” in the utterance “the door is not open” (Kaup, 2006; Liuzza et al., 2011; Bartoli et al., 2013; Foroni and Semin, 2013; García-Marco et al., 2019; Kaup and Dudschig, 2020; Marrero et al., 2020; Papitto et al., 2021; Montalti et al., 2023, 2024), several studies have demonstrated the involvement of right Inferior Frontal Gyrus in negation processing (de Vega et al., 2016; Papeo et al., 2016; Beltrán et al., 2018, 2019, 2021; Liu et al., 2020; Vitale et al., 2022). For example, inhibitory stimulation of rIFG by rTMS (repetitive transcranial magnetic stimulation) suppressed the inhibitory effect of negation on the action representation, equalizing motor excitability between negative and affirmative action sentences. This shows negation has a “disembodiment” effect that is reflected in reduced motor excitability during negated action comprehension (Liuzza et al., 2011; Bartoli et al., 2013; Papitto et al., 2021; Vitale et al., 2022).

Research has explored whether brain stimulation in the right temporal area, specifically in the medial aspects of the Superior Temporal Sulcus (STS), affects the processing of negation in attitudinal approach/avoidance sentences (Nuez et al., 2022). Participants read sentences such as: “Anne included (did not include) meat in her diet,” “Anne excluded (did not exclude) meat in her diet.” Then, 1,500 ms after the sentence display, they read a verb either mentioned in the sentence or its alternative (e.g., “include” or “exclude”). Results revealed that anodal stimulation increased availability of approach verbs (like “include”), indexed by shortened reading times. Additionally, this stimulation also made negated verbs more available. These findings suggest this right temporal area specializes in factual and approach information processing.

The so-called two-step model stands as the most widely accepted model to explain the processing of negation in language (Kaup, 2006; Kaup et al., 2006; Dudschig and Kaup, 2018; Kaup and Dudschig, 2020; Beltrán et al., 2021). According to this model (Kaup, 2006), comprehension of negative sentences happens in two sequential steps. First, negation focuses attention on what is negated (e.g., an “open door”). Following this, in a second step, this situation is dismissed, leading to an inhibited representation, evidenced by reduced accessibility of either an associated word or image in a recognition task. In this step, alternate scenarios (e.g., a closed door in the example above) might emerge. This is especially the case for binary categories (like even/odd) or contradictory terms (like open/closed) (Kaup et al., 2006). Within the two-step model framework, the effect of tDCS stimulation on medial rSTS in negation processing appears to occur in the first step, enhancing the effect of negation on availability of the negated element (Nuez et al., 2022; Vitale et al., 2022).

### 1.1 The present study

For the first time, we study the modulation of excitatory (anodal) tDCS in the rIFG on understanding approach/avoidance sentences in the context of negation. As in a previous experiment (Nuez et al., 2022), participants read attitudinal sentences either affirmative or negative. They then read a verb, either previously mentioned or its semantic alternative (e.g., “included” vs. “excluded”). We anticipated that brain stimulation of this area will enhance the inhibitory effect of negation. Specifically, we predict a specific modulation in the effect of negation in avoidance utterances. As avoidance verbs are implicitly negative, anodal stimulation will enhance the effect of the explicit negation, thus inhibiting the implicit negation, e.g.: NOT excluded = **NOT**→NOT (included). Thereby releasing the representation of the approach verb (“included”), making it more accessible. Anodal stimulation of the rIFG could facilitate the representation of the alternative (e.g., “included”) in negated avoidance sentences, whereas in approach sentences, it would inhibit the mentioned verb. (e.g., “included”). Yet, this representation is not necessarily inferred as in the two-step model.

### 1.2 Hypothesis

*Hl*: In the anodal condition, reading times for mentioned verbs will be longer in negative than affirmative sentences for both approach and avoidance, relative to the sham group.

*H2*: In the anodal condition, reading times for alternative verbs in avoidance negative sentences will be shorter than in approach negative sentences, with no difference in the sham group.

## 2 Method

### 2.1 Participants

Sixty-four university students from the University of La Laguna participated in the experiment, consisting of 46 women (71.87%) and 18 men (28.12%). The age of participants ranged from 18 to 25, and a 40-year-old participant, with a mean age of 21 years. Thirty-two participants were randomly assigned to the anodal stimulation condition, and 32 participants to the sham (placebo) condition. The sample size was calculated using G*Power (Faul et al., 2007) for a medium effect size *f* = 0.3, with a power of 0.8, and an alpha level of 0.05, for 2 groups with a total of 16 measurements, resulting in a required sample size of 52 participants. This sample size calculation was based on previous studies of our group with a similar design (Gámez and Marrero, 2001; Marrero et al., 2015, 2017, 2020, 2023a,b; Nuez et al., 2022).

### 2.2 Design

The study used a 2x2x2x2 experimental design with three within-subject factors: Target-Verb (mentioned vs. alternative), Sentence Direction (approach vs. avoidance) and Sentence Polarity (affirmative vs. negative). The between-subject factor was tDCS stimulation (anodal or sham). The dependent variable was the reading time of the target-verb.

### 2.3 Materials and stimuli

The experimental sentences underwent prior normative studies on the motivational direction (approach-avoidance). Controls were taken for lexical aspects such as sentence length and number of syllables, as well as psycholinguistic factors like sentence imaginability (Marrero et al., 2020). Target-verbs did not significantly differ either in length or in number of syllables, *p* > 0.10. The study used 140 sentences: 10 for each experimental condition and 60 fillers. The list of verbs was displayed in Marrero et al. (2020). Examples of the experimental sentences are shown in Table 1.

**Table 1:** Examples of sentences in both approach and avoidance versions.

Approach	Avoidance	Target
Julio**/ (no)** incluyó**/** la carne/ en la/ lista/ de la/ compra	Julio/ (no) excluyó/la carne/ de la/ lista/ de la/ compra	INCLUYÓ/EXCLUYÓ
Julio (**did not**) included meat in the shopping list	Julio/(did not) excluded meat in the shopping list	INCLUDED/EXCLUDED

The polarity manipulation is highlighted in bold between parentheses. The table also provides target verbs alongside their approximate English translations.

### 2.4 Procedure

Participants sat in front of a computer screen in an illuminated room and were instructed to read displayed sentences. First, they received instructions for task performance, and a training task with 8 example sentences. Before the tDCS application, participants read 30 test sentences to evaluation of differences in reading time between the anodal and sham groups before stimulation. Then, participants were submitted to 20 min of anodal tDCS, or 20 min of placebo stimulation (sham condition). After tDCS, they performed the experimental task.

**Figure 1:**
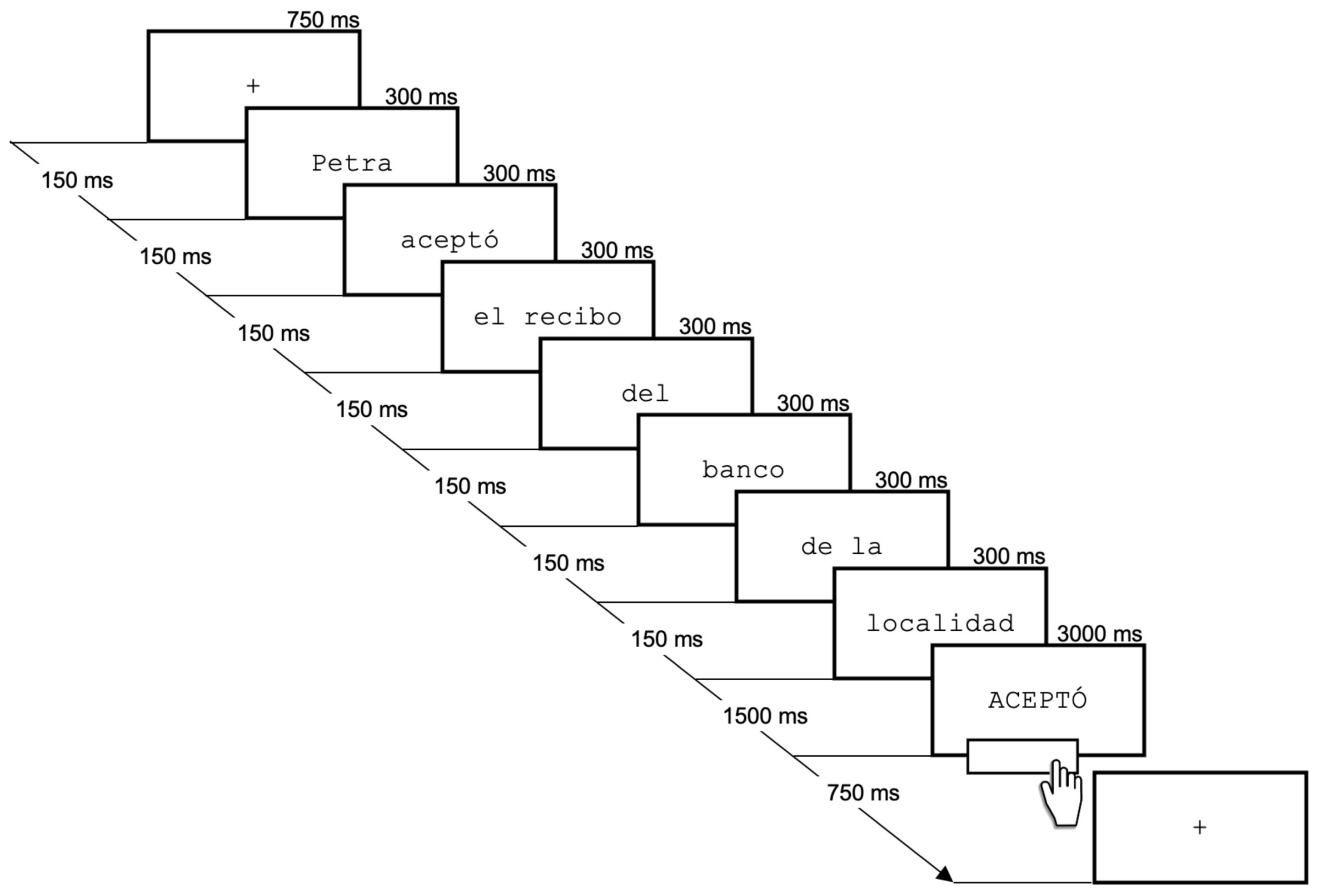
Presentation sequence of experimental sentences and the target (verb) with an example.

Each sentence presentation began with a central cross point displayed in the middle of the screen for 750 ms. followed by a 150 ms gap, and then a segmented sentence display, e.g., “Petra/accepted/the receipt/of the/bank/of the/town” *(“Petra/aceptó/el recibo/del/banco/de la/localidad”)* or its negative version: “Petra/did not accept/the receipt/of the/bank/of the/town” (*“Petra/no aceptó/el recibo/del/ banco/de la/localidad”*) (see Figure 1). A verb from the sentence (“accepted”) or its alternative (“rejected) appeared 1,500 ms post-sentence, which participants read and signaled completion by pressing the space bar. The verb remained on the screen for 3,000 ms or until a response was made. Participants were given 140 sentences, 10 for each experimental condition and 60 filler sentences. Filler sentences were thematically similar to experimental sentences with affirmative and negative versions.

To avoid participants focusing exclusively on the superficial reading of the target-verb, 25% of sentences (36 in total) were followed by content-related query, e.g., “Is it stated that Petra rejected the receipt of the bank?” Participants responded with YES (key “P”) or NO (key “Q”), with equal frequency for both options. The question remained on the screen for 5,000 ms or until a response was made. Feedback was shown for 2000 ms. After a delay of 750 ms, a new sentence was displayed. Participants were randomly assigned to one of the sets of sentences resulting from counterbalancing experimental conditions. This ensured that every participant received an equal number of sentences for each condition, and no participant received the same sentence twice. Sentence order within each set was random.

### 2.5 Transcranial direct current stimulation protocol

Transcranial direct current stimulation (tDCS) is a non-invasive brain stimulation tool that has shown great potential in improving cognitive performance. Studies highlight tDCS’s role in elucidating cortical substrates that underlie cognitive functions (Filmer et al., 2014). tDCS uses mild and constant electrical currents (typically up to 2 mA) to induce short-term changes in the excitability and cortical activation of the brain regions. Depending on current polarity, it can either excite or inhibit activity. Anodic tDCS increases the probability of firing action potentials via neuronal membrane depolarization, enhancing spontaneous activity in the targeted region and consequently functionally connected areas (Stagg et al., 2018). This highlights a causal relationship between cognitive functions and underlying cortical structures.

**Figure 2:**
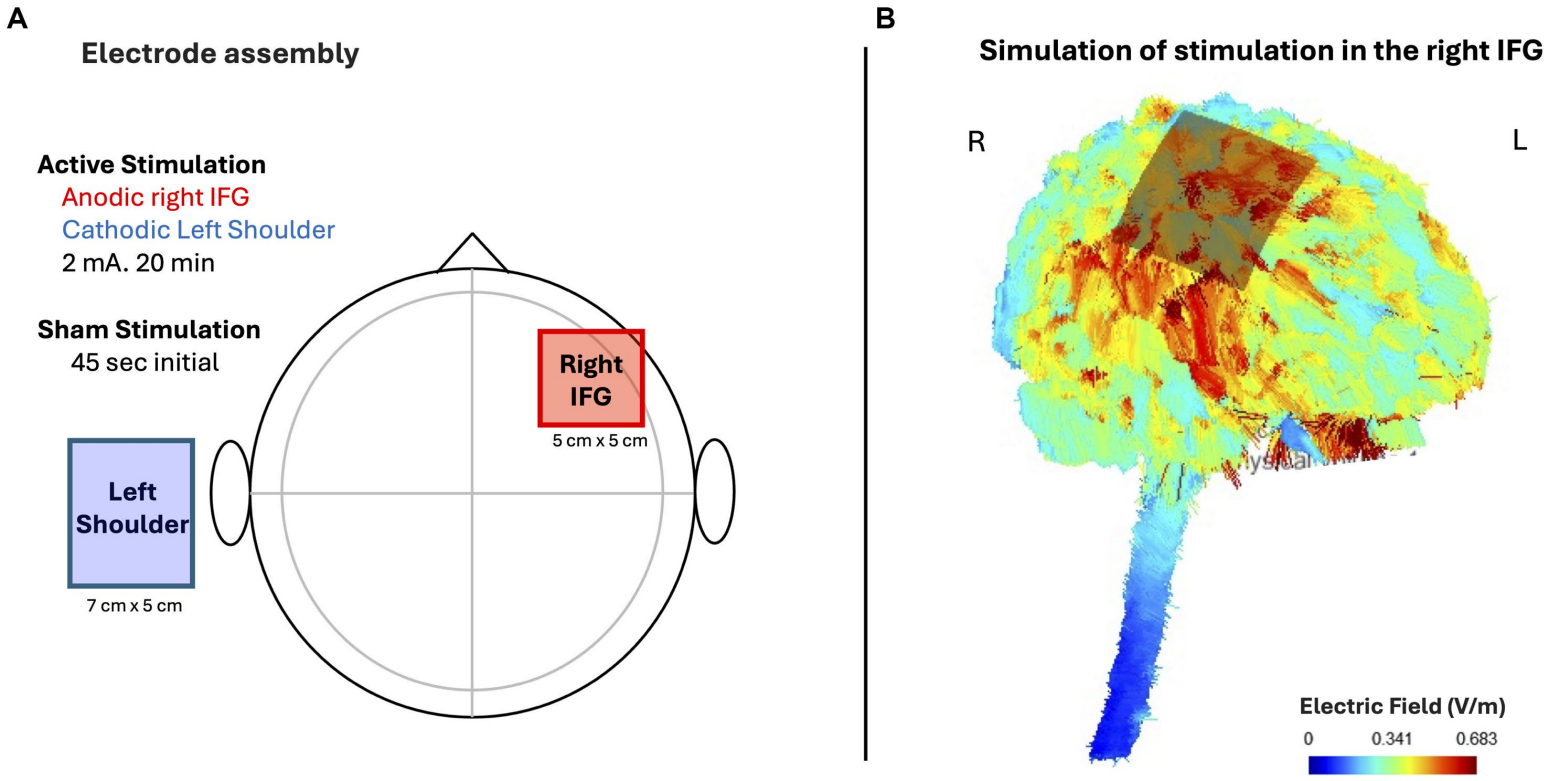
Simulation of electric field strength by SimNIBS 4 (Simulation of Non-invasive Brain Stimulation), of the active (anodal) electrode in the IFG. **(A)** Electrode assembly and **(B)** Simulation of stimulation in the right IFG.

For our study, a CE-certified battery-powered stimulator (neuroConn DCSTIMULATOR. neuroConn GmbH) was used for the non-invasive tDCS current conduction with an intensity at 2 mA. We used rubber electrodes of sizes 5 × 5 cm and 7 × 5 cm, covered saline-soaked sponges, yielding a density of 0.08 mA/cm^2^ and 0.057 mA/cm^2^, respectively. The smaller electrode was placed on the scalp in accordance with International System 10–20. The tDCS setup was positioned by connecting Fz to T4 and Cz to F8, intersecting the measurements in accordance with the international 10/20 system, which aligns with the rIFG. Our tDCS configuration targeted the right Inferior Frontal Gyrus (rIFG), as demonstrated in Figure 2 via SimNIBS 4 (Simulation of Non-invasive Brain Stimulation) software simulations (Thielscher et al., 2015). The stimulation application time was 20 min plus a 15 s fade-in and fade-out phases. The stimulation time was established based on previous studies of tDCS (Nitsche and Paulus, 2001). During the sham tDCS condition, participants followed the same procedure as the active stimulation and with the same electrode setup. The only difference was that the sham stimulation lasted only 45 s (fade-in for 15 s until reach the maximum intensity and fade-out for 15 s). During data collection, active stimulation and sham stimulation were alternatively administered.

#### 2.5.1 tDCS procedure

Upon entering the laboratory, participants were informed of the general objective of the study. Each participant completed a personal data form, a screening questionnaire for potential exclusion criteria, and signed a consent form. The participants were told that the aim of the study was to examine the effect of brain stimulation on cognitive improvement. The presence of a placebo (sham) condition was not disclosed to ensure participants remained unaware of the specific tDCS condition they were undergoing. None of them reported having epilepsy (or having close relatives affected), migraines, brain damage, heart disease, or other psychological or medical conditions and they were right-handed according to the Edinburgh Handedness Inventory (Oldfield, 1971). The ethical committee of the University of La Laguna approved the study: (CEIBA 2017–0272).

Next, participants performed the initial block of sentences of the reading task. Once the first block was completed, they were fitted with electrodes following the 20-min tDCS protocol. Directly after the tDCS equipment was removed post-session, participants performed the experimental sentence block. Once this task was completed, they were thanked for their collaboration and given a brief explanation of the study. Participants were asked not to discuss the experiment with other potential participants. The entire session lasted approximately 40 min. The stimulation parameters were considered safe (Bikson et al., 2016). We asked participants to report discomfort or any adverse side effects during tDCS (Brunoni et al., 2011; Kessler et al., 2012). Table 2 shows the adverse effects reported by the participants.

**Table 2:** Adverse effects, severity, and rounded percentage of participants that experienced them in the tDCS study.

Type of effect	Severity	% Anodal	% Sham
Headache	Mild	12.1%	19.4%
Neck pain	Mild	15.2%	6.5%
Scalp pain	Mild	12.1%	3.2%
Tingling	Mild	21.2%	45.2%
Itching	Moderate	12.1%	16.1%
Hot sensation	Mild	12.1%	9.7%
Reddening of the skin	Mild	24.2%	6.5%
Drowsiness	Mild	33.3%	19.4%
Concentration problems	Mild	24.2%	32.3%
Acute mood change	Mild	12.1%	3.2%

#### 2.5.2 COVID-19 protocol

Given the context of the ongoing COVID-19 pandemic during the time of the experiment, additional sanitary precautions were implemented. Every individual involved in the experiment, both participants and researchers, was mandated to sanitize their hands with hydroalcoholic gel, provided at the entrance prior to any activity. Wearing masks was obligatory for all. The cubicle used was thoroughly cleaned between each participant, with a special focus on frequently touched areas like the keyboard and mouse. Likewise, all tDCS-associated equipment, including measuring tapes, securing bands, electrode pads, and serum and gel syringes, were meticulously cleaned and sanitized after use.

### 2.6 Statistical analysis

First, we discarded any response times that were faster than 250 ms or were more than three standard deviations away from a participant’s average response time. Six participants (three each of Anodal and Sham group) were excluded due to more than half their trials exhibiting extreme slow response times. No other corrections were made on the data, following Linear mixed-effects models (LMMs) standards for data analysis (Scandola and Tidoni, 2021). Statistical analyses were conducted using (R Core Team, 2010). LMMs were implemented with the package lme4 (Bates et al., 2014). Contrast matrices were derived using the package hypr (Rabe et al., 2020; Schad et al., 2020), and model summary tables were generated using the package lmerOut (Alday, 2018). Based on our hypotheses, the outcome variable was analyzed for condition differences.

Response times were modeled as a function of the fixed terms Direction (approach vs. avoid), Polarity (affirmative vs. negative), Verb (alternative vs. mentioned), and inter tDCS factor (anodal vs. sham) and all higher-order interactions. For LMM modeling, the response time data were transformed to obtain the inverse Gaussian distribution, offering a better adjustment for right-skewed data compared to log-normal and Box-Cox distributions. Categorical variables were coded with sequential difference contrasts (for 2-level predictors 1/2, −1/2). Accordingly, the intercept was estimated as the overall mean of all conditions, and the resulting fixed-effects estimates can be interpreted as simple main effects based on the hypothesis matrix.

The model included random effects terms for individual participants and item intercepts. Following Scandola and Tidoni recommendations (Scandola and Tidoni, 2021), we applied an optimal trade-off between maximum random structure specification, convergence, and computational power in random effects specification and model selection. They highlighted the computational times are associated with convergence and overfitting problems. Thus, for cases of high model complexity - as is the case with our models - and relatively low computational power (standard laboratory equipment), they recommend employing Complex Random Intercepts (CRI). In a full CRI model, random (complex) slopes (with many interactions) are replaced by different random intercepts for each clustering factor, mitigating type I errors risks. For each analysis, we fit a maximum model. If the model did not converge, we removed the CRI that explained the least variance and tried again until a maximum model converged.

Coefficients with absolute *t*-values surpassing 1.96 in the model summaries were deemed as reliable estimates. The *t*-values greater than 1.96 can be considered approximate at the two-tailed significance level of 5%, since a *t*-distribution with a high degree of freedom approximates the *z*-distribution (Baayen et al., 2008).

In relation to the hypothesis we predict an interaction direction x polarity x verb x tDCS, and post-hoc comparisons support that (1) reading times for mentioned verbs will be longer in negative than affirmative sentences for both approach and avoidance, in anodal condition relative to the sham group, and (2) reading times for alternative verbs in avoidance negative sentences will be shorter than in approach negative sentences in anodal condition, with no difference in the sham group.

## 3 Results

A preliminary comparison of verb reading response times pre-tDCS stimulation revealed no significant disparities between the anodal and sham groups, with *p* > 0.10. A linear mixed model fitted by restricted maximum likelihood adjusted for the response time distribution was applied to the experimental data. The *t*-tests used the Satterthwaite method. We utilized the Holm method, a stepwise procedure designed to control the family-wise error rate in a conservative manner.

Although the four-way interaction direction x polarity x verb x tDCS did not reach a significance level (*β* = −0.23, *t* = −1.5), further examination of the predicted effects is pertinent to explore the interactions that arise separately for each type of tDCS stimulation (anodal vs. sham).

A summary of Linear mixed-effects model for the anodal condition is shown in Table 3.

**Table 3:** Summary of linear mixed-effects model for the anodal condition.

Variable	Estimate (β)	Std. Error	df	*t*	
Intercept	−1.60	0.098	38	*−17*	***
Direction	0.083	0.026	2,300	*3.2*	**
Polarity	−0.019	0.026	2,300	*−0.73*	
Verb	0.061	0.026	2,300	*2.4*	*
Direction * Polarity	−0.090	0.052	2,300	*−1.7*	**·**
Direction * Verb	0.077	0.052	2,300	*1.5*	
Polarity * Verb	0.140	0.052	2,300	*2.8*	**
Direction * Polarity * Verb	−0.300	0.100	2,300	*−2.9*	**

This table presents the fixed-effects estimates from our linear mixed-effects model for the anodal tDCS condition, detailing the main effects of Direction, Polarity, Verb, as well as their interactions on response times. The *t*-tests use Satterthwaite’s method and coefficients with absolute *t*-values greater than 1.96 are considered statistically significant at the *p* < 0.05 level. The significance levels are denoted as *p* < 0.1, ^*^p < 0.05, ^**^p < 0.01, ***p < 0.001.

Target-verb reading times across different conditions is shown in Table 4. We found a main effect of Direction (*β* = 0.083, *t* = 3.2); target-verbs were read faster in avoidance sentences (*M =* 769) than in approach sentences (*M* = 805). Additionally, we found a main effect of Verb (*β* = 0.061, *t* = 2.4); mentioned verbs showed shorter reading times (*M* = 766) than alternative verbs (*M* = 809). The polarity x verb interaction was significant (*β* = 0.14, *t* = 2.8). Post-hoc comparisons showed that mentioned verbs presented longer reading times in negative (*M* = 785) than in affirmative sentences (*M* = 747, *β* = −0.0912, SE = 0.0367, *z* = 2.482 = 2.4, *p* = 0.01). Conversely, in affirmative sentences, alternative verbs showed longer reading times (*M* = 825) than mentioned verbs (*M* = 747) (*β* = −0.1334, SE = 0.0367, *z* = 3.63, *p* = 0.003).

**Table 4:** Reading times (mean, standard deviation, and standard error) for target verbs across different conditions (direction and polarity) in the anodal group.

Stimulation	Direction	Polarity	Verb	*N*	Mean latency	Sd latency	SE latency
Anodal	Approach	Affirmative	Mentioned	293	756	419	24
Anodal	Approach	Affirmative	Alternative	291	828	480	28
Anodal	Approach	Negative	Mentioned	291	785	416	24
Anodal	Approach	Negative	Alternative	292	852	458	27
Anodal	Avoidance	Affirmative	Mentioned	287	739	408	24
Anodal	Avoidance	Affirmative	Alternative	289	822	428	25
Anodal	Avoidance	Negative	Mentioned	289	784	440	26
Anodal	Avoidance	Negative	Alternative	288	731	391	23

This interaction was qualified by a significant three-way direction x polarity x verb interaction (*β* = −0.3, *t* = −2.9). Pairwise comparisons showed that alternative verbs were read more slowly in approach negative sentences than in avoidance negative sentences (*β* = 0.2411, SE = 0.052, *z* = 4.64, *p* < 0.001). Alternative verbs showed longer reading times in avoidance affirmative sentences than in avoidance negative sentences (*β* = 0.1731, SE = 0.0521, *z* = 3.233, *p* < 0.001). The reading time of mentioned verbs was significantly shorter in avoidance affirmative sentences than in avoidance negative sentences (*β* = 0.1208, SE = 0.0521, *z* = −2.317, *p* = 0.02). Alternative verbs presented longer reading times than mentioned verbs in avoidance affirmative sentences (*β* = 0.1696, SE = 0.0521, *z* = 3.25, *p* = 0.001); and they presented longer reading times than the mentioned verbs in approach negative sentences (*β* = 0.1021, SE = 0.0518, *z* = 1.971, *p* = 0.0487). By contrast, alternative verbs presented shorter reading times than mentioned verbs in avoidance negative sentences (*β* = −0.1243, SE = 0.0521, *z* = 2.386, *p* = 0.0170). Neither the main effect of Polarity (*β* = −0.019, *t* = −0.73) nor the direction x verb interaction (*β* = 0.077, *t* = 1.5) were significant.

**Figure 3:**
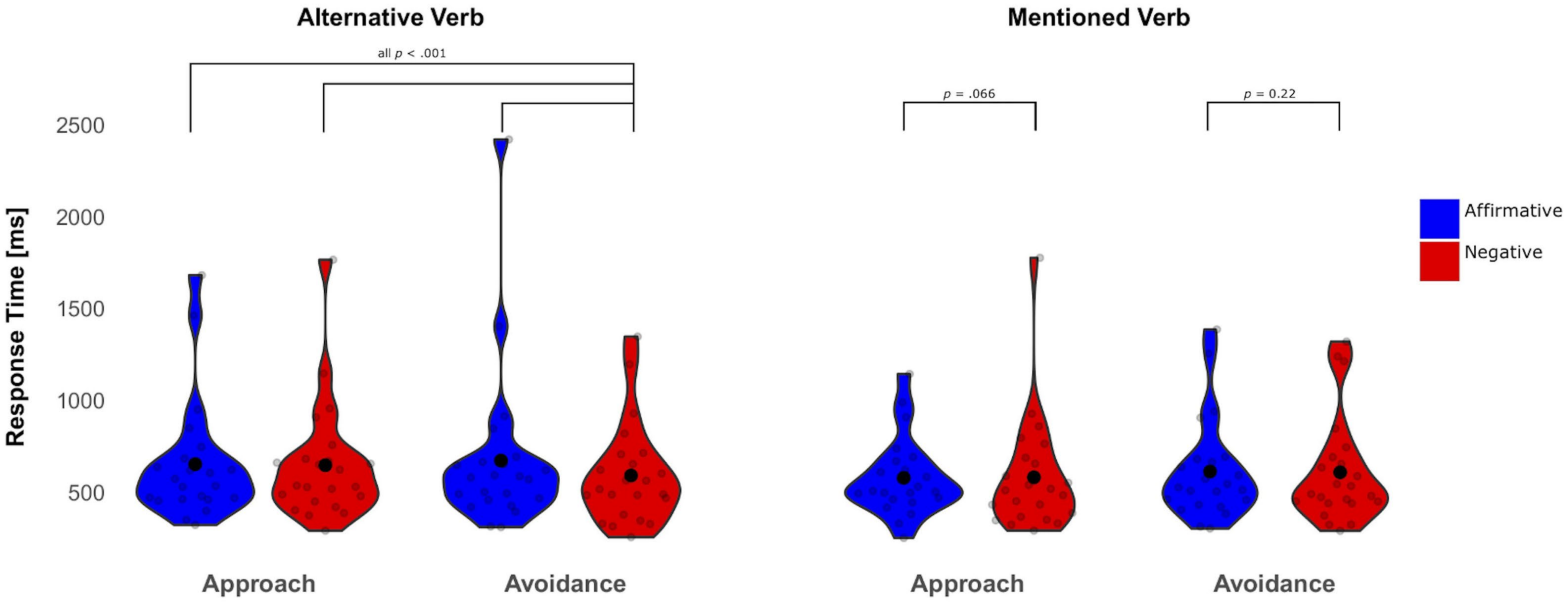
Violin and beeswarm plot of mean response times for alternative and mentioned verbs in approach and avoidance sentences as a function of polarity in the Anodal condition. Individual data points are represented as small circles, and the average for each condition is represented by a large black dot.

Figure 3 illustrates the effects of negation on reading times of mentioned verbs (a) and alternative verbs (b), which supports predictions from H1 and H2.

In the sham condition (Table 5), the triple interaction direction x polarity x verb was not significant (*β* = −0.069, *t* = −0.62). Similarly, none of the main effects of Direction (*β* = 0.0041, *t = 0*.1), Polarity (*β* = −0.044, *t* = −1.6), and Verb (*β* = 0.037, *t* = 1.3) were significant. In addition, the direction x polarity (*β* = 0.032, *t* = 0.58), direction x verb (*β* = 0.073, *t* = 1.), and polarity x verb (*β* = 0.014, *t* = 0.25) interactions were not significant.

**Table 5:** Reading times (mean, standard deviation, and standard error) for target verbs across different conditions (direction and polarity) in the sham group.

Stimulation	Direction	Polarity	Verb	*N*	Mean latency	Sd latency	SE latency
Sham	Approach	Affirmative	Mentioned	263	713	498	31
Sham	Approach	Affirmative	Alternative	262	728	464	29
Sham	Approach	Negative	Mentioned	268	699	455	28
Sham	Approach	Negative	Alternative	264	731	453	28
Sham	Avoidance	Affirmative	Mentioned	263	710	545	34
Sham	Avoidance	Affirmative	Alternative	264	754	568	35
Sham	Avoidance	Negative	Mentioned	263	710	462	28
Sham	Avoidance	Negative	Alternative	266	754	529	32

## 4 Discussion

Overall, the results support our hypotheses. In the anodal condition, the polarity x verb interaction supports H1, indicating longer reading times for mentioned verbs in negative than affirmative sentences. In the sham condition, there were no significant reading time differences for the mentioned verbs based on polarity. This suggests that anodal stimulation of the rIFG amplifies the inhibitory effect of negation for mentioned verbs. In addition, the three-way interaction direction x polarity x verb supports H2, alternative verbs in anodal condition resulted in shorter reading times in negative avoidance sentences compared to negative approach sentences.

These results contribute to our understanding of the brain’s basis of interaction of negation and approach-avoidance dynamics in linguistic and social communication. The different patterns of results between the anodal and sham conditions underline the rIFG pivotal role in the inhibitory effect over linguistic negation. The negation effect is different depending on whether the verbs are mentioned or alternative, and whether the sentences are approach or avoidance. For mentioned verbs, an amplified negation effect manifests through pronounced inhibition, indexed by less accessibility and longer verb-reading times, in negated sentences compared to affirmative sentences, either of approach or avoidance. This confirms our prediction for the second step of negation processing (Kaup, 2006; Kaup et al., 2006; Dudschig and Kaup, 2018; Beltrán et al., 2019; Kaup and Dudschig, 2020; Beltrán et al., 2021). For alternative verbs, the effect of negation depends on the direction of the verb, whether the sentence is of approach or avoidance. For avoidance verbs, anodal stimulation also enhances the effect of explicit negation, but in this case the effect is exerted on the implicit negation, e.g., NOT excluded = NOT→NOT (included), thereby releasing the representation of the alternative approach verb (“included”). This result suggests that the amplified inhibitory effect of negation, driven by right IFG brain stimulation, can be exerted over an implicit negation, enhancing the accessibility of the alternative scenario. However, its availability is not necessarily associated with inferring the implications of negation, as predicted by the two-step model.

Previous research has found that anodal tDCS stimulation of the right temporal lobe (around medial aspects of the STS) enhances the effect of negation through greater accessibility of what is negated (Nuez et al., 2022). If we compare the anodal stimulation in the right IFG with that of the right superior temporal sulcus, it suggests that negation processing could be performed in a sequential manner involving different brain areas, which could constitute a specialized brain network following the two-step model. The right temporal area would specialize in processing approach and factual nuances in language, and negation would be processed within the first step where attentional focus is on what is negated. In contrast, the right IFG would specialize in processing the negation function of inhibition, in the second step, which would give rise to a new and relevant effect in avoidance utterances where inhibition would be exerted in an implicit negation.

## 5 Contributions

This research explores the interplay between approach-avoidance language and negation processing and understanding the brain’s basis of these processes. Approach and avoidance attitudinal verbs play a pivotal role in the interwoven of language with interpersonal cognition (Tylén et al., 2010; Seyfarth and Cheney, 2014; Corballis, 2017), facilitating the communication of our own and others’ attitudes in social settings, and thereby regulating interpersonal interactions. Negation has long been recognized as an essential linguistic operator in social communication (Horn, 1989). The results of this study provide scientifically relevant knowledge in this regard.

Indeed, our results support the role of the rIFG in inhibition and negation processing from previous studies (de Vega et al., 2016; Papeo et al., 2016; Beltrán et al., 2018, 2019, 2021; Liu et al., 2020; Vitale et al., 2022). Moreover, we examine, for the first time, the communication of attitudes through language and its interaction with negation during comprehension. In particular, this study has investigated social avoidance in language where verbs are negatively implicit as well as highlighting the relevance of rIFG in avoidance processing.

## 6 Limitations

Stimulation with tDCS has certain limitations. For rIFG stimulation, we adhered to the 10/20 EEG system for electrode placement. However, factors such as inter-subject anatomical variability and the potential lack of focality in the applied stimulation could have influenced the results. Therefore, future research endeavors employing other techniques such as fMRI or TMS that could provide more direct and precise evidence their inhibitory role in negation processing. Also important, the four-way significant interaction did not result significant which weakens the contrast among anodal and sham group to support our hypothesis. A plausible explanation is that in the sham group we had not enough power. Future research is necessary for replication of the study by increasing samples, in particular of the sham group.

Our participant cohort predominantly comprised young, female university scholars. However, approach and avoidance brain encoding could be affected by developmental changes or be modulated by gender (Pintzinger et al., 2016; Torrence and Troup, 2018). Thus, future studies should also include a more heterogeneous demographic.

## 7 Conclusion

This study examines the effect of anodal tDCS on the rIFG in processing negation and social avoidance in language. Avoidance attitudinal verbs like “exclude” are implicitly negatively represented as “no-approach.” We found that anodal tDCS stimulation enhanced inhibitory effect of negation in two different ways. Firstly, longer reading times were shown in mentioned verbs in negated sentences compared to affirmative sentences. This result supports an inhibitory effect of negation on the verb mentioned, consistent with the second step of negation processing (Kaup, 2006; Kaup et al., 2006; Dudschig and Kaup, 2018; Kaup and Dudschig, 2020; Beltrán et al., 2021). Secondly, we found shorter reading times of alternative verbs in negative avoidance compared to negative approach sentences in anodal condition. This suggests that the enhanced inhibition from explicit negation was exerted over the implicit negation in avoidance sentences. To our knowledge, this provides novel evidence that an inhibitory effect of negation can be exerted on implicit negations, beyond its well-known effect on inhibition of narrated events. Moreover, this negation effect on a double negation highlights the role of rIFG in processing social avoidance meaning in language. Finally, we further theorize whether the rIFG might be part a brain network for processing the meaning of negation (Ghio et al., 2018) joined to right temporal area (specifically, medial aspects of the STS) that could specialize in the first step. Further research is necessary for examining this hypothesis.

## Data availability statement

The raw data supporting the conclusions of this article will be made available by the authors, without undue reservation.

## Ethics statement

The studies involving humans were approved by the ethical committee of the University of La Laguna approved the study: (CEIBA 2017-0272). The studies were conducted in accordance with the local legislation and institutional requirements. The participants provided their written informed consent to participate in this study.

## Author contributions

EG-M: Data curation, Formal analysis, Methodology, Writing – original draft, Writing – review & editing. AN: Project administration, Supervision, Writing – original draft, Writing – review & editing. IP: Data curation, Formal analysis, Methodology, Writing – original draft, Writing – review & editing. YR: Data curation, Writing – original draft, Writing – review & editing. YF: Writing – original draft, Writing – review & editing, Data curation, Formal analysis. HM: Project administration, Supervision, Writing – original draft, Writing – review & editing.
